# Determination of the size distribution of blood microparticles directly in plasma using atomic force microscopy and microfluidics

**DOI:** 10.1007/s10544-012-9642-y

**Published:** 2012-03-06

**Authors:** B. A. Ashcroft, J. de Sonneville, Y. Yuana, S. Osanto, R. Bertina, M. E. Kuil, T. H. Oosterkamp

**Affiliations:** 1 Leiden Institute of Physics, Niels Bohrweg 2, 2333 CA Leiden, The Netherlands; 2 Leiden Institute of Chemistry, Leiden University, Einsteinweg 55, 2333 CC Leiden, The Netherlands; 3 Department of Clinical Oncology, Leiden University Medical Centre, Albinusdreef 2, 2333 ZA Leiden, The Netherlands; 4 Einthoven Laboratory for Experimental Vascular Medicine and Department of Thrombosis and Haemostasis, Leiden University Medical Centre, Albinusdreef 2, 2333 ZA Leiden, The Netherlands

**Keywords:** Flow cell, AFM, PDMS, Microfluidics, Microparticles, Microvesicles

## Abstract

Microparticles, also known as microvesicles, found in blood plasma, urine, and most other body fluids, may serve as valuable biomarkers of diseases such as cardiovascular diseases, systemic inflammatory disease, thrombosis, and cancer. Unfortunately, the detection and quantification of microparticles are hampered by the microscopic size of these particles and their relatively low abundance in blood plasma. The use of a combination of microfluidics and atomic force microscopy to detect microparticles in blood plasma circumvents both problems. In this study, capture of a specific subset of microparticles directly from blood plasma on antibody-coated mica surface is demonstrated. The described method excludes isolation and washing steps to prepare microparticles, improves the detection sensitivity, and yields the size distribution of the captured particles. The majority of the captured particles have a size ranging from 30 to 90 nm, which is in good agreement with prior results obtained with microparticles immediately isolated from fresh plasma. Furthermore, the qualitative shape of the size distribution of microparticles is shown not to be affected by high-speed centrifugation or the use of the microfluidic circuit, demonstrating the relative stable nature of microparticles *ex vivo*.

**Electronic supplementary material:**

The online version of this article (doi:10.1007/s10544-012-9642-y) contains supplementary material, which is available to authorized users.

## Introduction

Blood microparticles (MPs), also known as microvesicles, are small particles shed from the surface of many cells upon stimulation or apoptosis (Diamant et al. 2004). For a long time they were considered as platelet dust (Wolf 1967), but now they have been recognized to participate in important biological processes (Cocucci et al. 2009). Examples of such processes are surface-membrane traffic and the horizontal transfer of protein and RNAs among neighboring cells, which are necessary for rapid phenotype adjustments in a variety of conditions (Cocucci et al. 2009). In addition, blood MPs have important physiological and pathological roles in blood coagulation, inflammation and tumor progression (Burnier et al. 2009; Pap et al. 2009).

Flow cytometry (FCM) and capture-based assays are commonly used methods to measure the number of MPs, define their origin based on membrane antigen expression, and asses their procoagulant features (Nomura et al. 2009; Lacroix et al. 2010; Aupeix et al. 1997). However, these methods have their drawbacks. FCM employs laser light which excites at 488 nm, while MPs may have sizes far below this wavelength (Furie and Furie 2006). Yuana et al. (2010) reported the presence of MPs bearing CD41 antigen in plasma with sizes ranging between 10–475 nm using atomic force microscopy (AFM). They also found that the MP numbers detected by AFM are 1,000-fold higher than those detected by FCM. Although capture-based assays using annexin V or MP-specific antibodies allow high throughput assessment of procoagulant features of MPs (Freyssinet 2003; Habib et al. 2008; Jy et al. 2004), these assays give no information on the size and total number of MPs in plasma.

Electron microscopy (EM) has been used for detection of MPs (Heijnen et al. 1999; Hughes et al. 2000; Aras et al. 2004), but this method only provides semi-quantitative information on MPs. Furthermore, sample dehydration and vacuum procedures required in EM might affect the characteristics of MPs. Recently, a promising method, nanoparticle tracking analysis (NTA), has been applied to count MPs in plasma (Harrison et al. 2009). This method uses a CCD camera system that allows simultaneous tracking of multiple particles. In the future NTA may be able to detect, count, and size antibody-labeled MPs efficiently, thus allowing the detection of subsets of MPs.

Not only is the analytical measurement of MPs a challenge, but also there is no golden standard yet to prepare MPs (Yuana et al. 2011). Many studies have isolated MPs from platelet free or platelet poor plasma by applying high speed centrifugation or even ultracentrifugation (Piccin et al. 2007; Enjeti et al. 2007). To prevent loss and phenotypic changes of MPs during the isolation procedure, using blood plasma directly for MP measurement would be preferable (Robert et al. 2009). Furthermore, the time between blood withdrawal and the actual MP test should be as short as possible to avoid activation of cells and coagulation processes which may affect MP numbers and characteristics.

We propose a method to detect MPs directly in blood plasma by using a microfluidic flow cell and performing subsequent analysis using AFM in liquid-tapping mode. Laminar flow patterns within the flow cell ensure complete fluid turnover in a controlled manner. The flow cell allows experimentation with very small sample volumes. In this study, a detachable flow cell was developed to enable direct contact between the fluid in the microfluidic channel and the surface. Diluted plasma was flown through the microfluidic channel with a controlled pressure driven laminar flow and made to be directly in contact with anti-CD41 antibody-coated mica. MPs exposing CD41 antigen were captured on this surface and subsequently imaged by AFM. We employed the AFM method for MP detection previously used in the study of Yuana et al. (2010).

Clotting of the plasma and clogging of the microfluidic channel pose a potential problem within such small volumes and with such a sensitive detection method as AFM. These problems have been solved by diluting plasma with either citrate or EDTA-enriched Hepes buffer and coating the microfluidics channels and the tubing of the microfluidics system. We demonstrated that this method increases the sensitivity of detecting specific MPs in a sample 100 to 1,000-fold. In conclusion, the application of a flow cell allows the AFM measurement of a specific subset of MPs directly in blood plasma.

## Materials and methods

### Blood collection and plasma preparation

After giving their informed consent, venous blood of three healthy volunteers is collected by using a 21-gauge needle (BD Vacutainer, San Jose, CA) with minimal stasis. Except for the first four ml, the blood is collected either in 1/10 volume of sodium citrate (3.2%, 0.105 M) or in K2 EDTA (3.6 mg) using 4.5 mL BD Vacutainer tubes (Becton Dickinson, San Jose, CA). Within 10–15 min after withdrawal, the collected blood is centrifuged at 2,000 *g* for 10 min at 20°C, without brake. The supernatant plasma is carefully collected and centrifuged again at 2,000 *g* for 10 min, 20°C, without brake, to obtain platelet poor plasma (PPP). PPP was aliquotted in 250 μL portions, snap frozen in liquid N_2_, and stored at −80°C until used. Before used, PPP is quickly frozen-thawed at 37°C. Unless stated otherwise PPP is used in the experiments.

### Microparticles isolation

For MP isolation, 750 μL of frozen-thawed citrate PPP is centrifuged at 18,890 *g* and 20°C for 30 min, with minimum brake. The supernatant is removed carefully, except for 25 μL containing the MP pellet. This pellet is resuspended in 1 mL of Hepes buffer [10 mM Hepes (Merck, Darmstad, Germany), 137 mM NaCl (Merck), 4 mM KCl (Merck), 0.1 mM Pefabloc SC (Fluka, Munich, Germany), pH 7.4], vortexed, and centrifuged as before. The supernatant is removed, leaving a volume of 25 μL containing the MP pellet. Subsequently, this 25 μL is carefully diluted with 725 μL of Hepes buffer to reconstitute to the original plasma volume (750 μL) before use in the experiment.

### Flow cell: mold fabrication

A flow cell mold is fabricated from brass. This brass is milled so that ridges with dimensions of 10 mm × 300 μm × 100 μm are created that shape the liquid channels during polymerization. The top surface of the ridges is polished to allow viewing through the channel from bottom to top after molding. At the end of the ridges, small holes are drilled and small pins are inserted with a diameter of 1 mm and a height of about 1 mm.

### Flow cell: fabrication

**Fig. 1:**
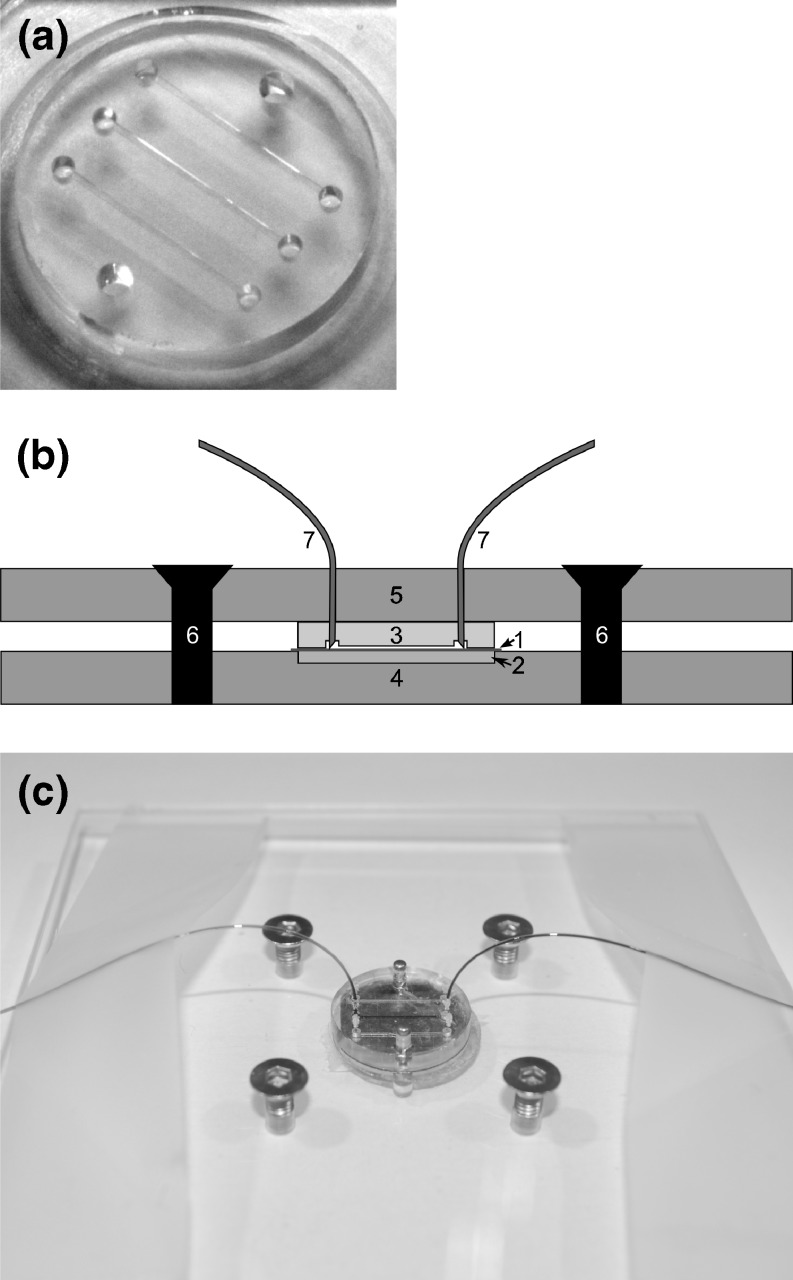
Flow cell setup. (**a**) Open PDMS flow cell. (**b**) Microfluidic flow cell setup with schematic side view. The holder system (4,5,6) is used to press the PDMS chip (3) onto the mica surface (1). The glass capillary tubes (7) are guided through holes in the holder top plate (5) to reach the connection chambers in the chip (3). The metal disc (2) is used as a support for the mica surface. In the photo (**c**), the middle channel is connected and filled with a dark blue solution; the glass capillary tubes are bent towards the side using scotch tape. The blue solution and scotch tape are for illustration purposes and are not used in experiments

Polydimethylsiloxane (PDMS) flow cells are fabricated using a Sylgard 184 kit (Dow Corning, UK). Silicone primer and catalyst are mixed in a 10:1 ratio by weight and this mixture is placed in a vacuum chamber for 1 h to remove air bubbles trapped during mixing. Next, the mixture is slowly poured into the mold and then the mold is carefully closed with a glass plate. The mold containing the polymer solution is placed in an oven at 70°C for 1 h. Afterwards, the glass slide with the PDMS flow cell is released from the mold and covered with a clean glass slide to keep the chip channel area dust-free. The polymerized flow cell is shown in Fig. 1(a).

### Flow cell: setup

The complete microfluidic setup is shown in Fig. 1(b). To prepare the flow cell setup, a mica surface (1) is placed on a metal support disc (2). The metal support disc is placed onto the bottom plate of the holder device (4), in a small cavity that closely fits the metal disc. The PDMS flow cell (3) is placed onto the top plate with the open microfluidic channels facing down. Two pins, situated in the holder top plate (5) align the flow cell (see the two holes next to the channels in Fig. 1(a)) with respect to the mica surface and the holes for the glass capillaries (7) (TSP Fused Silica Tubing, ID/OD 150/375 μm, deactivated with DPTMDS, from BGB Analytik Vertrieb, Germany). Then the top and bottom plate are pressed onto each other with four screws (6). Using microscopic inspection the screw pressure is carefully adjusted. The glass capillary tubes are beveled to 45° before use, using a mechanical grinder (Michael Deckel S0) with a disc containing diamond dust. After careful rinsing with water, to remove remaining grinding dust, the glass capillary tubes are gently forced into the PDMS flow cell, and guided through alignment holes situated in the holder top plate.

### Mica surface preparation for attachment of anti-human CD41 monoclonal antibody

The surface of mica (Electron Microscopy Sciences, Washington) for MP attachment is prepared as described before (Yuana et al. 2010) with a slight modification. Freshly cleaved mica disks (diameter 12 mm) are overnight immersed in DMSO containing 55% (w/v) ethanolamine at 70°C. Subsequently, the mica surfaces are rinsed twice with dry DMSO at 70°C and then with HPLC grade ethanol to remove the DMSO. Next, the mica surfaces are put for 10 min into 30 mL phosphate buffered potassium (PBK) (10.2 g KCl, 0.97 g K_2_H_2_PO_4_ and 5.71 g K_2_HPO_4_ per L) (pH 7.4) previously saturated with EGTA by adding 100 mg EGTA. The surfaces are then rinsed with Hepes buffer, before 20 μl of 0.05 mg/mL (in Hepes buffer) mouse anti-human CD41 antibody clone P2 (Beckman Coulter, Fullerton, CA) is applied to the surface and incubated for 3 h. Excess anti-CD41 is removed by washing with Hepes buffer. Anti-CD41 antibody coated-mica surfaces are stored in Hepes buffer until used. As a negative control, mouse IgG1 pure clone X40 (Becton Dickinson, San Jose, CA) is used (0.05 mg/mL in Hepes buffer). The IgG1 isotype control antibody is allowed to incubate for 3 h on the functionalized mica surfaces. All chemicals are purchased from Sigma Aldrich (Munich, Germany) unless otherwise indicated.

Prior to the attachment of MPs antibody-coated mica surfaces were inspected by using AFM to ensure that the number of false spots and holes in the antibody coating was minimized.

### Attachment of microparticles without using microfluidics

PPP (100 μL EDTA plasma) is dropped onto the mica surface coated with IgG1 isotype control and anti-CD41 antibody (“drop method”). To check the saturation of MPs on the anti-CD41-coated surface, PPP is incubated on the surfaces for 2, 30, and 60 min. Similar to what was found by Yuana et al*.* (2010), 30 min incubation seemed to be sufficient. On anti-IgG1-coated surface PPP was incubated for 60 min to match the long exposure time on the anti-CD41 surface. The surfaces are carefully rinsed with Hepes buffer and then scanned by AFM to determine the number of MPs captured on CD41- and IgG1 isotype control- coated mica surfaces.

### Attachment of microparticles using microfluidic flow cell

The open microfluidic flow cell (PDMS) is attached to a mica surface as described above. A 1 mL-syringe (Becton Dickinson, San Jose, CA, USA) is connected to a Harvard Apparatus PicoPlus (Harvard apparatus, Holliston, MA, USA) syringe pump and set at a constant flow speed of 0.01 mL/min. The syringe is connected to the glass capillary tubes using Luer-Lock Adapters and One-Piece Fittings from LabSmith (Livermore, CA, USA). The glass capillary tubes are gently forced into the microfluidic chip using the beveled end.

The channels of the flow cell are rinsed with 50 μl EGTA-enriched Hepes buffer (5 mM EGTA, 10 mM Hepes, 137 mM NaCl, 4 mM KCl, 0.1 mM Pefabloc® SC, pH 7.4) buffer for about 5 min. Hundred fifty μL of either EDTA plasma diluted with EDTA-enriched Hepes buffer (20 mM EDTA (Sigma Aldrich), 10 mM Hepes, 137 mM NaCl, 4 mM KCl, 0.1 mM Pefabloc® SC, pH 7.4) or isolated MPs diluted with Hepes buffer is allowed to flow through the channel in the flow cell for about 15 min total flow time. The channel is then rinsed with 50 μL Hepes buffer (~5 min flow time). Before removal from the flow cell, the back of the mica is carefully marked to indicate the location of the channel in the AFM. Subsequently, the flow cell is removed and the coated surface with the attached MPs is rinsed with Hepes buffer and stored in Hepes buffer until imaged by AFM. All steps are performed at room temperature (RT).

### AFM imaging

AFM imaging is performed with a Digital Instruments Multi-mode AFM (Veeco, New York, NY, USA) using the E scanner. Olympus cantilevers (Olympus, Tokyo, Japan) with force constant of 2 N/m and a resonant frequency of 70 kHz are used. The liquid cell tip holder (Veeco) is rinsed with ethanol and milli-Q water between each sample to prevent contamination. Each image was scanned at 10 × 10 μm and 10 images are taken at a variety of locations on the surface. For each particle, the sum of pixel heights multiplied by the pixel area is used to estimate a volume and subsequently to calculate its (spherical) diameter.

## Results

**Fig. 2:**
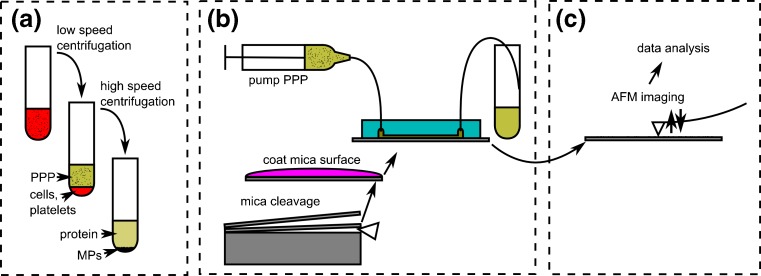
Schematic overview of experiments. Collected blood plasma is centrifuged twice to acquire PPP. In some experiments blood plasma proteins are removed by use of high-speed centrifugation (**a**). Using an antibody coated mica surface, a fresh PDMS chip and a holder system a microfluidic setup is build, and the PPP is run over a small surface area (**b**). Finally, mica surface is removed and imaged using AFM, followed by automated image analysis (**c**)

Generally, glass, polymer or similar materials are used with microfluidics. However, the AFM cantilever must have physical access to the top of the sample and AFM requires an atomically flat background to give the best image of the sample. Mica is preferred surface material because it has distinct atomically flat layers that can be easily separated for cleaning and functionalization. As PDMS binds strongly to mica, the mica surface can be pealed away when the PDMS is removed, ruining the sample. In our study, the mica surface is functionalized with antibodies to produce a hydrophilic mica surface that cannot bind to the PDMS flow cell. Anti human-CD41 antibody was used to coat the functionalized mica to capture platelet MPs (PMPs) bearing CD41 surface antigen. PMPs constitute 80–95% of blood MPs detected by FCM (Horstman and Ahn 1999; Tesselaar et al. 2007). The IgG1 isotype control is used as a control for nonspecific binding of MPs on anti-CD41-coated surface. A schematic overview of the experiment is given in Fig. 2. The microfluidic setup used in Fig. 2(b) is constructed from a PDMS flowcell (Fig. 1(b)), attached to the mica surface by a removable holder system (Fig. 1(b,c)) so that the flow cell can be removed from the surface of the sample without damaging either the attached MPs or the mica.

### Application of microfluidic system to count MPs in plasma

With microfluidics many more MPs in the plasma sample will have a chance to interact with the antibody-coated mica surface by flowing an equal volume of plasma over a very small active surface area in the confined volume of the microfluidic channel. To examine this we applied the microfluidic system and compared it with the drop method to count MPs in plasma obtained from two healthy donors. For the microfluidic system two samples were prepared: the first sample consists of MPs isolated from citrate PPP, reconstituted to the original plasma volume, and subsequently diluted 5 times with Hepes buffer; the second sample is EDTA PPP diluted 5 times with EDTA-enriched Hepes buffer. For the drop method undiluted EDTA plasma is used.

In all samples processed by the microfluidic system, the AFM detected MPs attached on anti-CD41-coated mica surface (Table 1). The attachment of these MPs was specific because there were at least two times less particles found attached on the IgG1-coated surface compared to those on the anti-CD41-coated surface (Table 1). This also confirms that these MPs bear CD41 surface antigen (CD41-positive MPs). Using image quantification software we found in the samples processed by microfluidic system that there is no significant difference in the number of MPs attached on anti-CD41-coated surface (218, 276, 203, and 240 MPs/100 μm^2^). Strikingly, there were hardly any MPs captured on anti-CD41 coated-surface by using the drop method, even after one hour incubation of plasma on the surface (3 MPs/100 μm^2^ anti-CD41-coated surface and 3 MPs/100 μm^2^ IgG1-coated surface, Table 1).

**Table 1:** Comparison of microfluidic method and drop method to count CD41-positive MPs. For the microfluidic method MPs isolated from citrate PPP and diluted EDTA PPP of two healthy donors, D1 and D2, were used. Isolated MPs were reconstituted to the original plasma volume and subsequently five-fold diluted in Hepes buffer (Reconstituted isolated-MPs D1/D2). EDTA PPP was diluted five-fold with EDTA-enriched Hepes buffer (EDTA plasma D1/D2). For the drop method undiluted EDTA PPP from the same healthy donors was used (EDTA plasma drop D1/D2)

Samples	Number of particles attached on anti-CD41-coated mica		Number of particles attached on IgG1 isotype control-coated mica	
	Mean	SEM	Mean	SEM
Reconstituted isolated-MPs D1	218	51	82	22
Reconstituted isolated-MPs D2	276	50	1.7	0.6
EDTA plasma D1	203	80	70	19
EDTA plasma D2	240	40	1.8	0.04
EDTA plasma drop D1	0.15	0.09	2	0.55
EDTA plasma drop D2	3	1.5	3	1.0

It has been reported in the literature (Gachet et al. 1993; Rao et al. 1997) that when EDTA is used as anticoagulant for blood collection, the CD41/CD61 complex on the plasma membrane of platelets may loose their affinity for anti-CD41 and CD61 antibodies. To check this we have used FCM to measure the binding of anti-CD61 and anti-CD41 antibodies to platelet MPs in citrate- and EDTA-anticoagulated blood plasma. We found that the numbers of CD41/CD61-positive MPs in citrate and EDTA plasma are not significantly different (see Figure S1 in the supplementary information).

Despite the fact that macroscopic clotting could be prevented by diluting EDTA PPP with EDTA-enriched Hepes buffer, we noticed in preliminary experiments that there still was some clotting in the small confinements of the microfluidic channel. In some studies it has been shown that unmodified PDMS is not compatible with some of the blood/plasma components and may initiate activation of the clotting system (Belanger and Marois 2001; Whitlock et al. 1999). Platelets adhere more strongly to the surface of unmodified PDMS than to the modified PDMS (Khorasani and Mirzadeh 2004). Moreover, unmodified PDMS is hydrophobic and this might induce clotting when plasma is introduced in the microfluidic channel (Thorslund et al. 2005a; Thorslund et al. 2005b). To prevent this clotting in the microfluidic channels, a solution of EGTA-enriched Hepes buffer was flowed through the channel before application of the plasma. EGTA is also known as a strong chelator of calcium ions, but it is not known to the authors whether the EGTA also can physically be adsorbed on the PDMS, acting to prevent clotting on the surface of the channel, or if EGTA performs its anti-clotting action in some other way.

### Analysis of AFM images

**Fig. 3:**
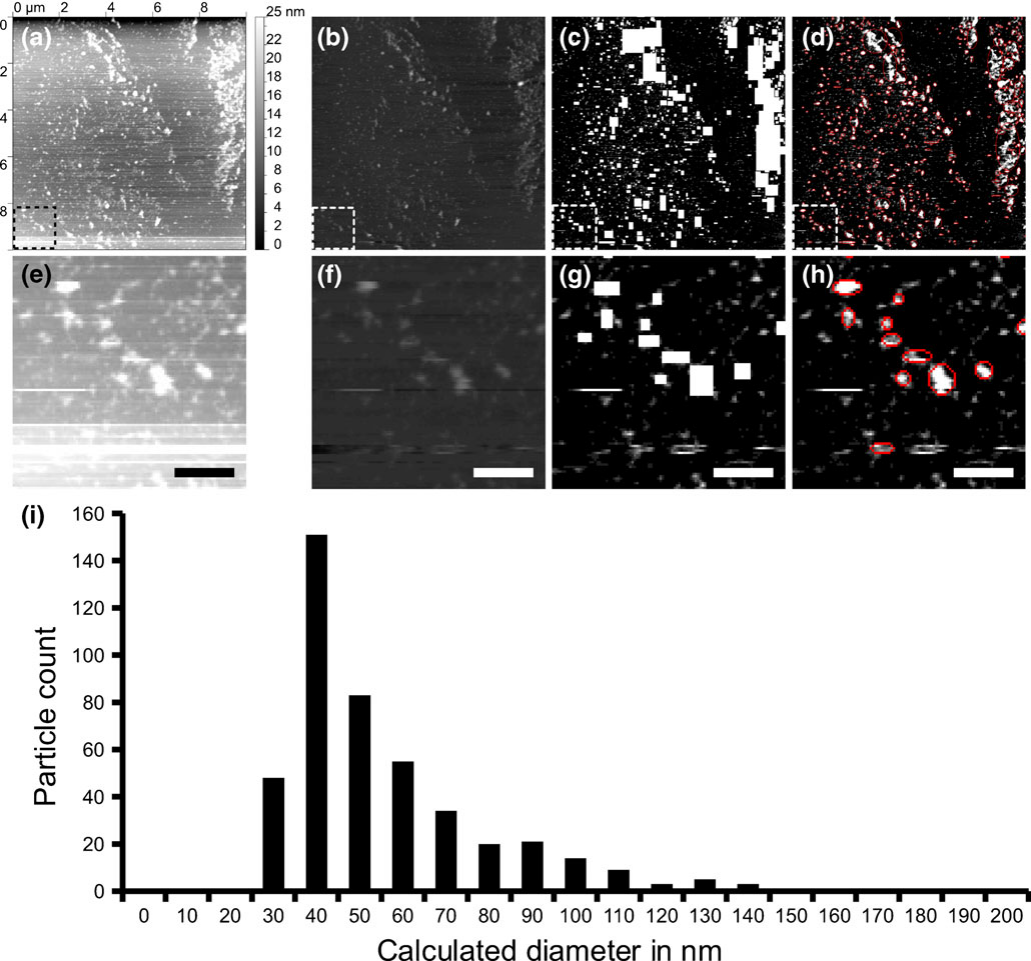
AFM image quantification. Original AFM image, intensity represent height, see scale bar on the right side (**a**). This image is flattened using the standard linear regression background subtraction (**b**). White squares show all the particles that are found on the image from image a (**c**). The background is subtracted, corrected for the shadowed regions, and the particles are correctly sized (**d**). The bottom row (**e**, **f**, **g**, **h**) shows an enlarged region of (**a**, **b**, **c**, **d**) respectively, scale is 500 nm. The measured particles are indicated with red ellipses (**d**, **h**). The size distribution graph of particles detected from this image (100 μm2) is depicted (**i**)

The AFM images provide a unique challenge for image processing. As images are generated by scanning line after line, each neighboring line scan can have a different offset, slope or parabolic background (Fig. 3(a,e)). This background must be dealt with for the accurate determination of neighboring scan lines to calculate the heights and volumes of the MPs correctly. The most commonly used techniques involve performing linear regression on the fast scan line and then subtracting the background estimate from each line. This technique is frequently foiled by small, high features on the surface, such as MPs. To overcome this difficulty, a special technique is developed in our lab. First, a standard linear regression subtraction is performed (Fig. 3(b,f)). Second, Labview IMAQ is used to find all the particles (Fig. 3(c,g)). Third, the regions containing the particles are then removed from the background subtraction input and the linear regression subtraction is performed again to provide a much flatter surface (Fig. 3(d,h)). While this is a computationally expensive task, it provides the high precision background subtraction for the needed accurate determination of the particle volumes.

Particle counting is performed by using the Labview IMAQ library to determine the location of the particles. The Imaq Count Objects 2 VI is first used to filter and obtain a list of possible particles. This software uses a threshold to make a binary image, and then uses the watershed method to count the particles. Particles that touch the boundaries will not be counted. Additionally, all holes within the particles are automatically filled. The lower limit of height, width, and breadth of the particles is set to separate them from the background. For counting, the particle must at least 3 nm high and occupies at least 3 pixels. The particle must be larger than 1 pixel in width or breadth. By setting this limit constant selection rules can be applied throughout a large dataset of AFM images.

As can be deduced from Fig. 3 the height is smaller than the width in detected MP profiles, the MPs appear disc shaped after binding to the surface. Using the disc radius and height, the particle volume is estimated and converted into an effective diameter assuming that MP have a spherical shape in solution (Yuana et al. 2010). This procedure ignores the effects of tip flattening. It seems that this does not have a major effect on the final calculated volume of the particles (results not shown), and only a small systematic error exists in the volume calculations from the tip broadening effect. The size distribution of the example image is shown in Fig. 3(i).

Size distribution graphs are made to further analyze possible differences between MPs captured from diluted isolated MPs and from diluted EDTA plasma, to see if high-speed centrifugation has an effect on the particle size. No significant differences in the number and size distribution of CD41-positive MPs was observed before and after high-speed centrifugation (supplementary Table S1, supplementary Figure S2). As mentioned before, we do not need to concentrate particles using high-speed centrifugation, however the clotting probability is strongly reduced by removal of blood plasma proteins. Therefore we use purified MPs, reconstituted to the original volume for all further experiments.

### Relationship between MP concentration and number of MPs captured on anti-CD41-coated surface

**Fig. 4:**
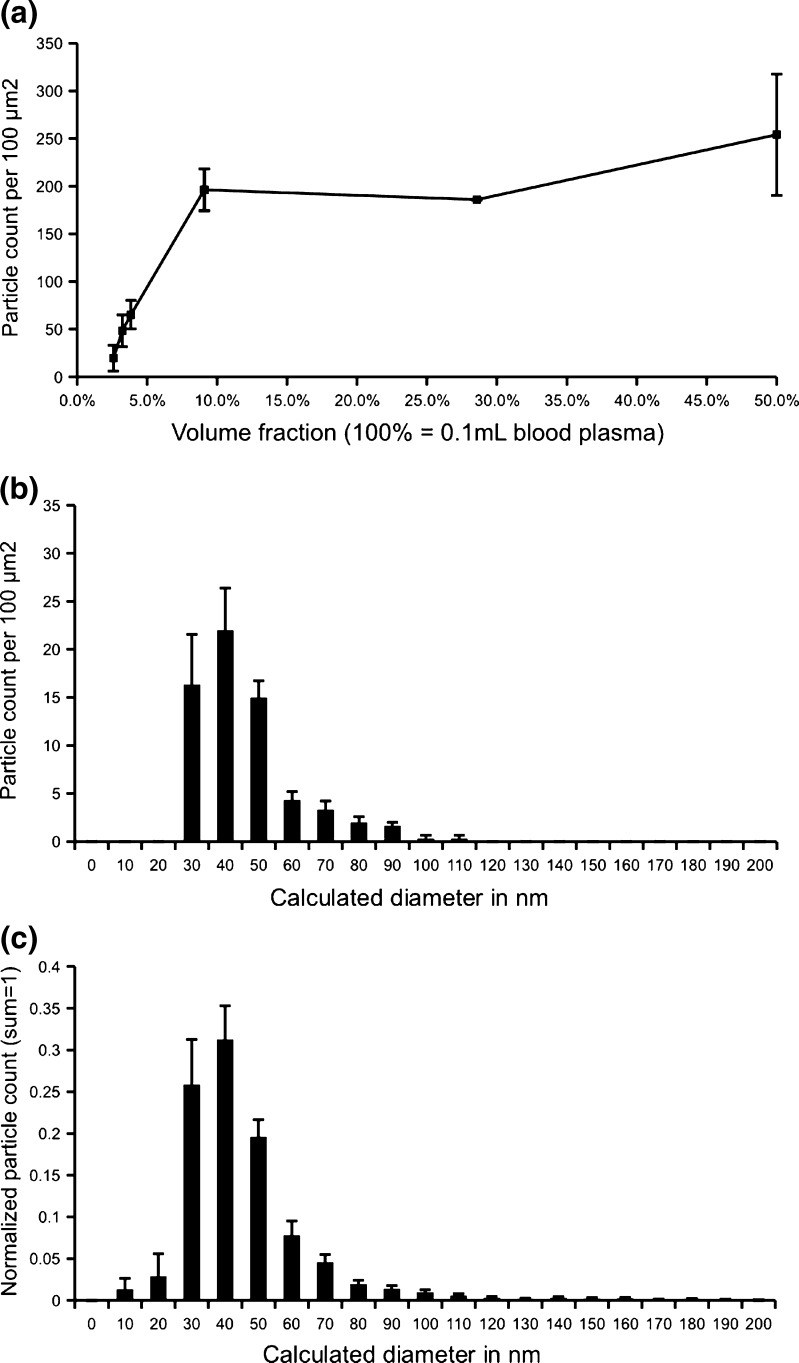
Relationship between the MP concentration in the sample and the number of CD41-positive MPs detected by AFM. MPs isolated from frozen-thawed citrate PPP are diluted from 50% to 2.5% (100% is undiluted reconstituted-isolated MPs) in Hepes buffer and run through the microfluidics device (**a**). These experiments were done on two different days using the same plasma pool of one healthy volunteer. The size distribution from a single dilution (3.8%) is based on three images (**b**). A normalized size distribution of all dilutions averages is weighted equally (**c**). Scale bars represent the standard error of the mean

Prior to measuring the concentration of MPs in a sample, the dynamic range should first be established. Therefore we used MPs isolated from frozen-thawed citrate PPP of a healthy volunteer. Isolated MPs are first reconstituted with Hepes buffer to reach the original plasma volume before isolation (100%) and subsequently diluted 2 to 40-fold. These diluted MP fractions are run through the microfluidics system to measure the number of captured CD41-positive MPs. Figure 4(a) shows that only at sufficiently low concentrations (<10%) there is a linear relationship between the MP concentration and the number of particles attached on the anti-CD41-coated surface. Probably because of unspecified binding in the microfluidics circuit the line does not cross the origin (0,0). At higher concentrations (>10%) the number of MPs captured to the anti-CD41-coated surface reaches a maximum of ~250 particles/100 μm^2^. This number of captured MPs is very similar to those reported in Table 1 for the reconstituted isolated MP fraction and EDTA plasma which are diluted 5 times before processing with the microfluidic system.

The AFM images show that, typically, the particles are not uniformly distributed on the surface (Fig. 3(a)). As a result, the standard error of the mean is quite large (Fig. 4(a)). The linear range is rather small, and it may be difficult to find a suitable working range when samples differ as strongly in particle counts as mentioned before (Yuana et al. 2010). Interestingly, it is found that the size distribution does not differ significantly between different images (Fig. 4(b)), and different dilutions (Fig. 4(c), Supplement Table 2) of the same sample, with the exception of the highest dilution. This sets microfluidic capture combined with AFM imaging as the first method able to measure the size distribution of a specific subset of MPs directly in PPP.

## Discussion

We report on a novel method to identify and characterize a specific subset of MPs directly in plasma. To enable the measurement of MPs directly in plasma, we have developed a method that combines a microfluidic system and AFM detection. Microfluidic channels allow blood plasma to flow over a small surface of antibody coated mica, resulting in a high enough surface concentration of specifically bound MPs to detect and quantify using AFM. A much higher number of MPs is captured from (diluted) plasma on antibody-coated mica than without, using this microfluidic method (plasma drop system). Further optimization of the method is required for high-throughput measurements.

For the first time it is demonstrated that the size distribution of CD41-positive MPs is robust against high-speed centrifugation and dilution. However, to prevent clotting to occur, the use of MPs isolated by means of high-speed centrifugation is advised.

Yuana et al*.* (Yuana et al. 2010) have shown that MPs isolated from fresh citrate PPP have calculated, spherical like diameters (*d*_*sph*_) of ~50 nm (range 10–475 nm). Using microfluidics we found that MPs isolated from frozen-thawed citrate PPP and frozen-thawed EDTA PPP have a similar calculated diameter (*d*_*sph*_) of ~45 nm. Software has been developed to automate the measurement and counting of MPs. This results in much more consistent results and provides faster data analysis. By comparing the results from previous experiments by Yuana et al*.* (Yuana et al. 2010) to the new automated quantification of the same dataset we observed that the size distribution was similar to those reported earlier (data not shown).

There are advantages and disadvantages in using the microfluidic system and AFM to measure MPs. In this study we found that about 10 μL of plasma is enough to count significant number of MPs and determine their size distribution. Furthermore, the microfluidic system allows the measurement of MPs directly in plasma thus reducing time between venepuncture and MP measurement and also preventing MP loss because of washing steps in the isolation procedure. However, the preparation of mica (modification and coating) typically takes two days. In 20% of all cases we also dealt with leakage from microfluidic channels during plasma injection. AFM scanning of the surface is also time-consuming. It takes at least an hour finding a right surface to position the AFM tip and scanning 10 images of 100 μm^2^ at different locations on the surface.

Therefore, when this present method will be used as a diagnostic tool, the throughput needs to be increased by developing high speed AFM and automated sample handling. Additionally, if fluorescent labeling can be implemented efficiently, the number of particles captured should allow optical detection of these particles by means of fluorescence imaging. With the AFM technique being used for calibration, it should be possible to fluorescently tag the MPs to provide optical quantification of the number of MPs on the mica surface.

## Conclusion

In this study, it is demonstrated that by using a removable microfluidic circuit, CD41-positive MPs can be captured directly from diluted blood plasma, and detected by AFM. Quantification of MPs is automated, to allow consistent and fast quantification. Use of the microfluidic system increases the sensitivity of MP detection considerably, leading to a higher surface concentration of attached MPs, reducing the AFM scanning time. Direct use of plasma as opposed to isolated MPs shortens the pre-processing time and enables the detection of MPs in a more natural state. Ten μL EDTA plasma is sufficient to quantify the number and determine the size distribution and shape of CD41-positive MPs using microfluidics and AFM.

In future experiments the characterization of MPs from other origins (endothelial cells, monocytes, tumor cells, etc.) by using antigen-specific monoclonal antibodies will be addressed. This will help in monitoring subsets of MPs that may play a specific role in the development of certain diseases.

## Electronic supplementary material

Below is the link to the electronic supplementary material.

Supplementary Table 1 Statistics obtained from comparing distributions of isolated MPs against plasma MPs (see supplementary Figure 2). (DOCX 15 kb)

Supplementary Table 2 Statistics obtained from the dilution experiment described in section 3.3 (DOCX 14 kb)

Supplementary Fig. 1 Comparison of CD41/CD61-positive MPs in citrate and EDTA plasma. EDTA and citrate PPP were isolated from blood of a healthy volunteer. EDTA and citrate PPP are assayed fresh, after storage for 2 h at room temperature (RT), after frozen-thawed at 37°C, and after frozen-thawed at 37°C and stored for 2 h at RT. These PPP samples were directly stained by using PE-labeled anti-CD41 and FITC-labeled anti-CD61. The number of CD61/CD41-positive MPs was measured by FCM. All experiments were performed in duplicate. (TIFF 270 kb)

Supplementary Fig. 2 Distributions of the effective diameter of CD41-positive MPs. Black bars show the size distribution of particles captured from MPs isolated from frozen-thawed citrate PPP. Grey bars show sizes of particles captured from frozen-thawed EDTA PPP diluted five-fold with EDTA-enriched Hepes buffer. All counts are normalized so the sum of the probabilities is 1. See also Supplementary Table 1. (TIFF 188 kb)
