# Supplementary material for: Determination of the size distribution of blood microparticles directly in plasma using atomic force microscopy and microfluidics
Source: Biomed Microdevices. 2012 Mar 6;14(4):641–9. doi: 10.1007/s10544-012-9642-y (PMC3388260; doi:10.1007/s10544-012-9642-y)
Supplement: Supplementary file 1 — Statistics obtained from comparing distributions of isolated MPs against plasma MPs (see supplementary Figure 2). (DOCX 15 kb) [file 10544_2012_9642_MOESM1_ESM.docx]

**Supplementary Table 1** Statistics obtained from comparing distributions of isolated MPs against plasma MPs (see supplementary Figure 2).

|  | Plasma | Purified MPs |
| --- | --- | --- |
| Number of processed images | 8 | 9 |
| Number of particles | 1992 | 527 |
| Mean* | 58 | 63 |
| Median* | 52 | 50 |
| Standard deviation* | 23 | 43 |
| Range* | 223 | 367 |
| Min* | 26 | 23 |
| Max* | 249 | 390 |

*Values based on calculated particle diameter in nanometer
