# Supplementary material for: Determination of the size distribution of blood microparticles directly in plasma using atomic force microscopy and microfluidics
Source: Biomed Microdevices. 2012 Mar 6;14(4):641–9. doi: 10.1007/s10544-012-9642-y (PMC3388260; doi:10.1007/s10544-012-9642-y)
Supplement: Supplementary file 2 — Statistics obtained from the dilution experiment described in section 3.3 (DOCX 14 kb) [file 10544_2012_9642_MOESM2_ESM.docx]

**Supplementary Table 2** Statistics obtained from the dilution experiment described in section 3.3

| Percent Reconstituted MPs | 2.6% | 3.2% | 3.8% | 9.1% | 29% | 50% | 80% |
| --- | --- | --- | --- | --- | --- | --- | --- |
| Dilution | 75 | 60 | 50 | 20 | 5 | 2 | 1 |
| Number of processed images | 5 | 7 | 3 | 9 | 1 | 5 | 5 |
| Number of particles | 98 | 339 | 196 | 1766 | 186 | 1270 | 373 |
| Mean* | 30 | 49 | 46 | 54 | 50 | 45 | 51 |
| Median* | 29 | 42 | 42 | 46 | 46 | 40 | 41 |
| Standard deviation* | 12 | 27 | 15 | 28 | 20 | 17 | 34 |
| Range* | 68 | 192 | 87 | 301 | 152 | 160 | 283 |
| Min* | 8 | 26 | 27 | 26 | 26 | 25 | 23 |
| Max* | 76 | 218 | 114 | 327 | 177 | 185 | 306 |

*Values based on calculated particle diameter in nanometer
