# Supplementary figures and images for: Determination of the size distribution of blood microparticles directly in plasma using atomic force microscopy and microfluidics
Source: Biomed Microdevices. 2012 Mar 6;14(4):641–9. doi: 10.1007/s10544-012-9642-y (PMC3388260; doi:10.1007/s10544-012-9642-y)

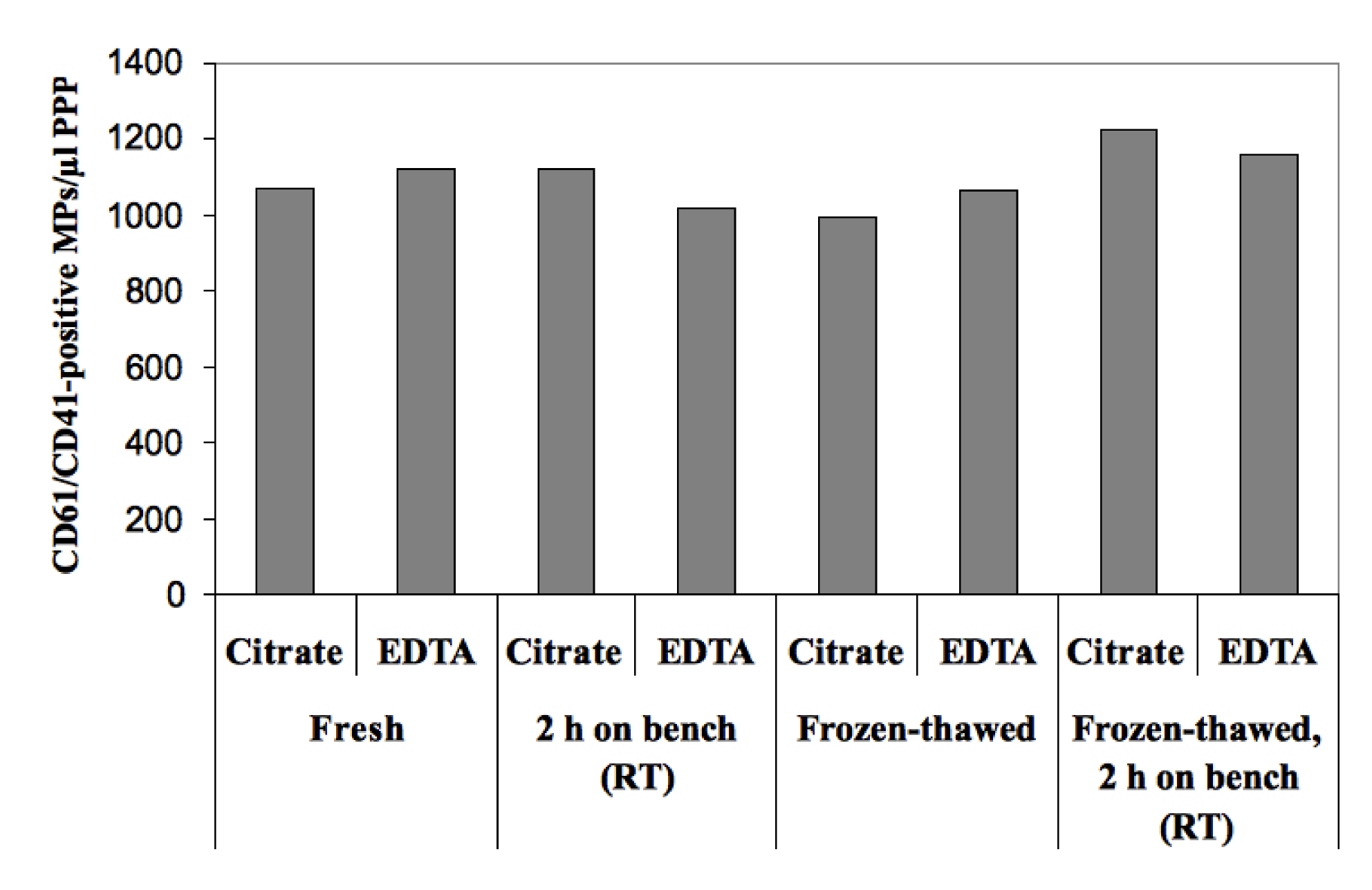

Supplement: Supplementary file 3 — Comparison of CD41/CD61-positive MPs in citrate and EDTA plasma. EDTA and citrate PPP were isolated from blood of a healthy volunteer. EDTA and citrate PPP are assayed fresh, after storage for 2 h at room temperature (RT), after frozen-thawed at 37°C, and after frozen-thawed at 37°C and stored for 2 h at RT. These PPP samples were directly stained by using PE-labeled anti-CD41 and FITC-labeled anti-CD61. The number of CD61/CD41-positive MPs was measured by FCM. All experiments were performed in duplicate. (TIFF 270 kb) [file 10544_2012_9642_MOESM3_ESM.tif]

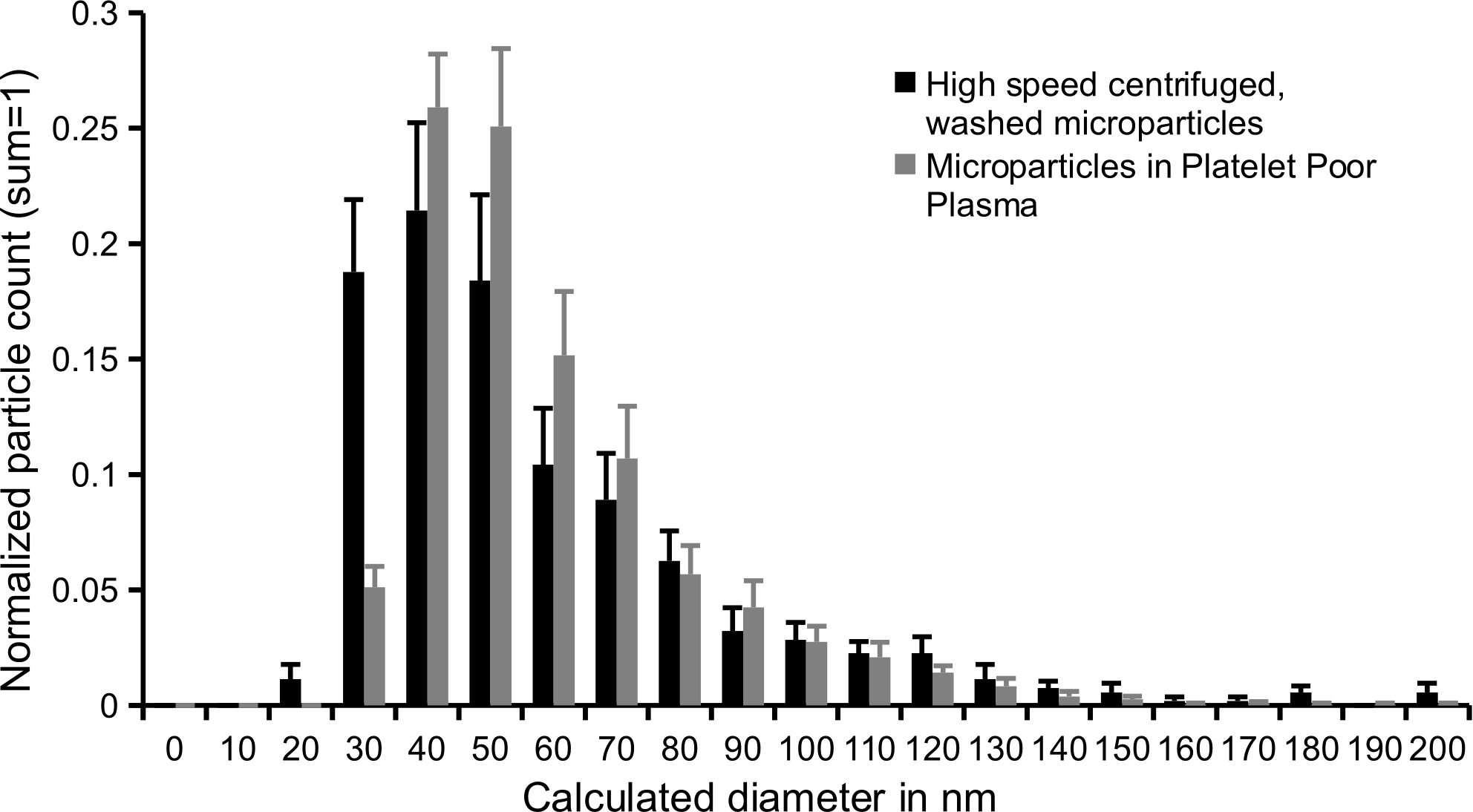

Supplement: Supplementary file 4 — Distributions of the effective diameter of CD41-positive MPs. Black bars show the size distribution of particles captured from MPs isolated from frozen-thawed citrate PPP. Grey bars show sizes of particles captured from frozen-thawed EDTA PPP diluted five-fold with EDTA-enriched Hepes buffer. All counts are normalized so the sum of the probabilities is 1. See also Supplementary Table 1. (TIFF 188 kb) [file 10544_2012_9642_MOESM4_ESM.tif]
